# How structural racism, neighborhood deprivation, and maternal characteristics contribute to inequities in birth outcomes

**DOI:** 10.1093/haschl/qxae092

**Published:** 2024-07-23

**Authors:** Anuj Gangopadhyaya, Lisa Dubay, Emily Johnston, Vincent Pancini

**Affiliations:** Department of Economics, Quinlan School of Business, Loyola University, Chicago, IL 60611United States; The Urban Institute, Health Policy Center, Washington, DC 20034, United States; The Urban Institute, Health Policy Center, Washington, DC 20034, United States; The Urban Institute, Health Policy Center, Washington, DC 20034, United States

**Keywords:** birth outcomes, racial disparities, structural racism

## Abstract

Decades of disparities in health between infants born to Black and White mothers have persisted in recent years, despite policy initiatives to improve maternal and reproductive health for Black mothers. Although scholars have increasingly recognized the critical role that structural racism plays in driving health outcomes for Black people, measurement of this relationship remains challenging. This study examines trends in preterm birth and low birth weight between 2007 and 2018 separately for births to Black and White mothers. Using a multivariate regression model, we evaluated potential factors, including an index of racialized disadvantage as well as community- and individual-level factors that serve as proxy measures for structural racism, that may contribute to White–Black differences in infant health. Finally, we assessed whether unequal effects of these factors may explain differences in birth outcomes. We found that differences in the effects of these factors appear to explain about half of the underlying disparity in infant health.

## Introduction

In the United States, there are large and persistent racial disparities in birth outcomes, with high rates of adverse outcomes found among births to non-Hispanic Black mothers. In 2021, rates of preterm birth ranged from 14.75% for births to non-Hispanic Black mothers to 9.5% for births to non-Hispanic White mothers.^1^ In the same year, the rate of low birth weight among non-Hispanic Black mothers was twice as high as that for non-Hispanic White mothers (14.66% compared with 7.03%).^1^ Both preterm birth and low birth weight can have physical, developmental, and economic costs to infants and their families at birth and over the life course.^2,3^

Disparities in infant and maternal health between non-Hispanic Black (hereafter referred to as Black) and non-Hispanic White (hereafter referred to as White) mothers are pervasive and not immediately attributable to differences in socioeconomic status. For instance, infants born to Black mothers at the top of the income distribution experience markedly worse outcomes than infants born to White mothers at the bottom of the income distribution.^4^ These disparities have persisted throughout the 20th and 21st centuries.^5^ Today, these disparities remain despite initiatives to improve maternal and infant health outcomes for Black mothers and major changes to national health policy such as the Affordable Care Act (ACA).^6^

Prior research has identified a range of possible contributors to poor birth outcomes for Black mothers and infants, from differences in individual-level socioeconomic factors to systemic factors including racism, discriminatory policies, chronic stress, weathering (the accelerated deterioration of health over time due to the cumulative effects of racism), and environmental stress.^7-11^ There is no support for the theory that differences in adverse birth outcomes are driven by biological differences between Black and White mothers.^12^

Despite increased awareness of the role that structural racism plays in poor health outcomes for Black people, measurement of this relationship is challenging. The effects of racism are pervasive throughout US society at institutionalized, personally mediated, and internalized levels and in policies, practices, and values of all types.^13,14^ Moreover, there is no 1 measure that captures the extent of structural racism in a place.

A small but growing body of literature has used index-based measures of structural racism to investigate this relationship. One such measure, the Index of Concentration at the Extremes (ICE), measures the extent to which a geographically defined population is concentrated into relative extremes of advantage and deprivation.^15^ A racialized version of ICE has been used to study the relationship between structural racism and health outcomes, typically defining White households with high incomes as advantaged and Black households with low incomes as disadvantaged and classifying counties as relatively advantaged or disadvantaged based on the difference in the shares of the population these groups represent in each area. State-specific studies using ICE have found a strong negative association between ICE and adverse birth outcomes, documenting that outcomes for Black mothers and their infants improve as an area's privilege increases and its deprivation falls.^16,17^ Another index-based measure, the Neighborhood Deprivation Index (NDI), measures neighborhood-level wealth, income, education, employment, and housing—domains shaped by racist and classist policies and their effects. The NDI may, therefore, serve as a measure of structural racism in ways that are not fully captured by the ICE measure. Few studies in this growing literature have used multiple index-based measures to encompass the extensive effects of structurally racist practices and policies on infant health.

To better understand how structural racism drives racial differences in adverse birth outcomes, we examined trends in rates of preterm birth and low birth weight between 2007 and 2018 at different levels of racialized disadvantage as measured by ICE, NDI, and maternal-level factors that can also be influenced by structurally racist institutions and policies. We used multivariate regression and a Kitigawa-Oaxaca-Blinder decomposition analysis to understand the contributions of differences in racialized economic segregation, neighborhood disadvantage, and maternal characteristics to racial differences in adverse birth outcomes. We further examined whether there are unequal effects of these factors on birth outcomes by race.

This study strengthens the evidence base on factors that contribute to the large White–Black disparity in infant preterm birth and low birth weight. It is the first national assessment of how racialized economic segregation is contributing to disparities in infant outcomes by race, as prior studies using ICE were state-specific analyses.^14,16^ Second, we extended our analysis beyond a single measure of structural racism, ICE, to further consider the impacts of neighborhood deprivation and maternal characteristics on infant birth outcomes. Finally, we considered not only the impact of differences in mutable characteristics between White and Black mothers but also the impact of differences in the effects of these characteristics by race on birth outcomes. This analysis indicates whether, for example, Black and White mothers residing in a more affluent or less-deprived neighborhood experience the same protection against adverse birth outcomes.

Assessing this research question is critical to understanding the extent to which policies, which are often designed to achieve equal levels of characteristics but typically ignore heterogeneous effects of these characteristics, may succeed in establishing equitable infant health outcomes.

## Data and methods

### Conceptual framework

Structural racism and classism are fundamental causes of poor health; they privilege some groups and communities and disadvantage others by differentially distributing access to power, justice, employment opportunities, income and wealth, education, health-promoting neighborhood infrastructure, and health care.^18-24^ These structural forces operate through policies, laws, values, discrimination, and implicit bias, and they result, through multiple pathways, in unequal health status for Black and other people of color and people with low-incomes.^18,22,25-27^

Structural inequities, such as those driven by racial and economic segregation, determine who has access to health-promoting opportunities, such as safe neighborhoods, affordable housing, well-resourced schools, quality jobs, low levels of police violence and incarceration, and culturally appropriate health care.^14,24,27-30^

In this paper we focus on residential segregation and neighborhood deprivation, measured by ICE and NDI, respectively. We hypothesized that those residing in neighborhoods with high relative concentrations of advantaged high-income White households will benefit from greater neighborhood-level opportunities, while those residing in the most disadvantaged neighborhoods will lack these resources, leading to worse infant outcomes. Further, we hypothesized that those residing in areas with greater deprivation will be more likely to experience adverse infant outcomes.

Combined with societal structural factors, these community-level inequities, in turn, influence individuals' opportunities for education, income and wealth, and marriage. We consider these maternal characteristics in our models as the downstream effects of structural racism that may directly affect infant outcomes. Together, these societal, community, and individual inequities combine to produce health disparities through multiple mechanisms, including chronic stress, weathering, and environmental exposures.^7-11^

### Data and measures

We used restricted-access vital statistics natality data from all registered birth certificates from 2007–2018, limiting our analysis to singleton births. We focused on pre-pandemic years to better isolate the association between structural racism and infant health outcomes. These data provide information on maternal, infant, and delivery characteristics and identify mothers' state and county of residence. We identified the share of births that were preterm (gestational length at or earlier than 37 weeks) and the share of births with low birth weight (<2500 g). We used parental racial-ethnic information reported on birth certificates to identify White and Black mothers.^31^

We used ICE, NDI, and maternal-level characteristics as our proxy measures of structural racism. Consistent with prior literature, we specified our ICE measure to define advantaged populations as White households with incomes in the top quintile of the national income distribution and disadvantaged populations as Black households with incomes in the bottom quintile of the income distribution. Throughout this analysis, we used 2006–2010 American Community Survey data to identify these populations by county; this is approximately consistent with the baseline years for this analysis.

The ICE measure ranges from −1 to 1 with −1, indicating an area in which the entire population is composed of low-income Black households and 1 indicating an area in which the entire population is composed of high-income White households. Using each county's ICE measure, we categorized counties into 5 quintiles, with the lowest quintiles representing the counties with the highest relative concentration of low-income Black household and the highest quintiles representing the counties with the highest relative concentration of advantaged high-income White households.

The NDI measures county-level deprivation based on wealth, income, education, employment, and housing.^32^ We obtained the NDI for the years 2006–2010 from a publicly available R package (R Foundation for Statistical Computing).^33^ As with our ICE measures, we grouped each county into quintiles of the NDI measure, with lower quintiles representing counties with less neighborhood disadvantage and higher quintiles representing counties with the greatest neighborhood disadvantage. We present the distributions of both our ICE and NDI measures in Figure S1.

We investigated the role of maternal factors available on the birth certificate, including mothers' age at birth, mothers' education (high school or less education, some college education, Bachelor's or greater education, or education status missing), mothers' marital status (married, not married, or marital status missing), and birth parity (first birth or second or later birth).

### Methods

We first examined trends in preterm birth and low birth weight between 2007 and 2018 separately for births to Black and White mothers and trends in the White–Black difference for each outcome. We further examined trends in birth outcomes by the ICE quintile of the mother's county of residence, considering outcomes separately for births to Black and White mothers and trends in the White–Black difference for each outcome.

We next used multivariate regression analysis to assess how ICE, NDI, state of residence, and maternal characteristics affect the overall difference (ie, total disparity) in birth outcomes between infants born to White and Black mothers across all analysis years. We clustered standard errors at the county-level, the level at which ICE and NDI vary.

Finally, we decomposed the extent to which differences in outcomes by race can be attributed to differences in the characteristics of Black and White mothers and the counties in which they reside or, instead, by differences in the effects of these characteristics on birth outcomes among Black and White mothers. For example, living in a county with a higher concentration of advantaged households (or higher ICE values) may benefit infants born to White mothers more than infants born to Black mothers. We implemented a Kitigawa-Oaxaca-Blinder decomposition, which evaluates the share of the overall White–Black difference in infant birth outcomes attributable to White–Black differences in the level of observed factors (the measures included in our multivariate regression models described above) and the share that can be attributed to differences in the effects of these factors (ie, coefficients) on birth outcomes.^34^

Our study has several limitations. First, our analysis is descriptive and consequently we cannot make causal inferences about our results. Causal analyses of structural racism are complicated empirically because many proxy measures, such as ICE, can be viewed as both independent and dependent variables.^35^ Second, neither ICE, NDI, nor maternal characteristics, independently or together, are likely to capture the full effects of structural racism on health. As a result, our regression approach likely omits variables that describe structural racism and therefore likely underestimates the encompassing effects of structural racism on infant health disparities. Third, we measured ICE at the county level because that is the smallest geographic level that is available on the birth certificate files, but measuring ICE at a smaller geographic level, such as the census tract, may be more appropriate. Fourth, we focused on White–Black differences in birth outcomes and have ignored disparities between White women and infants and other women and infants of color who face structural racism.

## Results

Figure S2 plots rates of preterm birth and low birth weight from 2007 to 2018 among infants born to Black mothers and, separately, infants born to White mothers and additionally plots the White–Black differences in these outcomes. Relative to infants born to White mothers, infants born to Black mothers had much higher rates of preterm birth and low birth weight throughout this period. In fact, in every year, infants born to Black mothers were more than twice as likely as those born to White mothers to be low birth weight. Figure S2 is consistent with White–Black differences in infant outcomes reported in the literature.^36,37^

**Figure 1.**
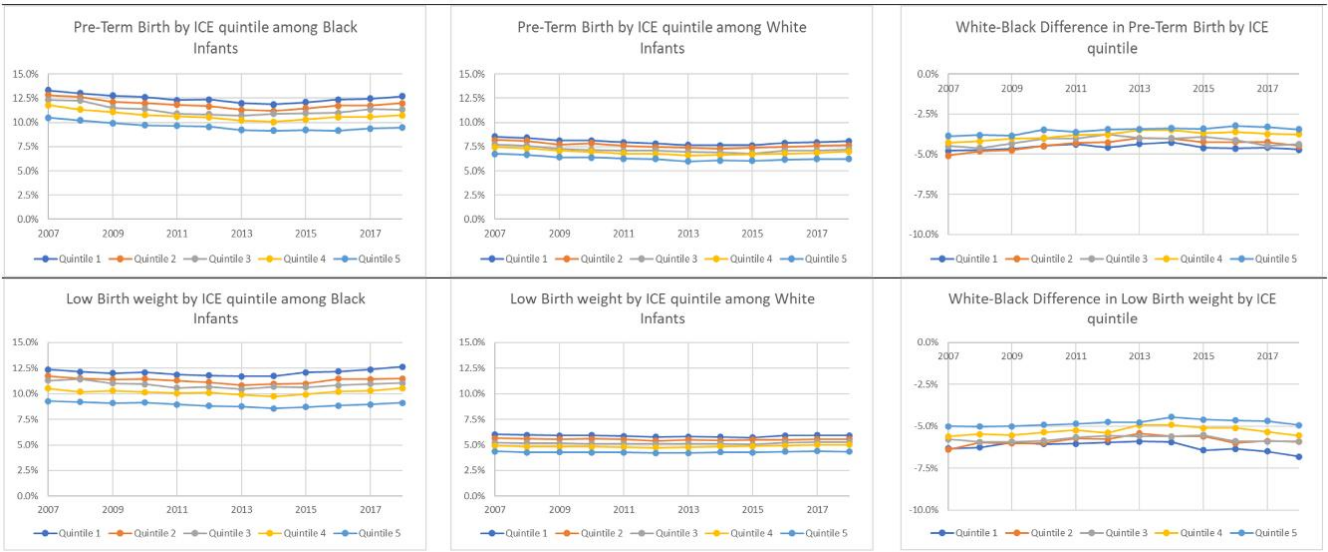
2007–2018 share of births born preterm or with low birth weight among infants born to Black and White mothers, by ICE quintile. Source: 2007–2018 Vital Statistics Natality Files. ICE quintiles were constructed from 2006–2010 American Community Survey files, designating advantaged populations as non-Hispanic White households with incomes in the top income quintile and disadvantaged populations as non-Hispanic Black households with incomes in the bottom income quintile. Preterm birth was defined as births with obstetric estimates of gestational length at or earlier than 37 weeks. Low birth weight was designated based on birth weight <2500 g. Abbreviation: ICE, Index of Concentration at the Extremes.

We evaluated whether birth outcomes for infants born to Black and White mothers vary by the quintiles of ICE (Figure 1). Counties with higher ICE quintiles are more advantaged and those with lower ICE quintiles are more disadvantaged.

Among infants born to Black mothers, Figure 1 shows a clear separation of birth outcomes by ICE quintiles. In all years 2007–2018, infants born to Black mothers residing in counties in the fifth quintile (ie, most advantaged counties) had the lowest rates of preterm birth and low birth weight. Conversely, infants born to Black mothers residing in the lowest or second-lowest ICE quintiles consistently had the highest rates of preterm birth and low birth weight. For example, in 2018, 12.6% of Black infants in quintile 1 were born preterm compared with 9.7% of Black infants in quintile 5.

Infants born to White mothers residing in counties with the most advantaged ICE quintile had the lowest rates of preterm birth and low birth weight relative to all other infants born to White mothers, and those in counties with the most disadvantaged ICE quintile had the highest rates of adverse birth outcomes. Absolute differences in the rates of preterm birth and low birth weight across ICE quintiles are much narrower among infants born to White mothers than for those born to Black mothers. For example, in 2018, 5.8% of infants born to White mothers residing in quintile 1 were low birth weight compared with 4.4% of those in the highest quintile counties. However, relative differences are comparable between racial groups, with a relative difference between the highest and lowest quintiles of 32% for infants born to White mothers and 30% for infants born to Black mothers.

In all years, infants born to Black mothers residing in the most advantaged counties (quintile 5) had higher rates of preterm birth and low birth weight than infants born to White mothers residing in the most disadvantaged counties (quintile 1).

We further investigated differences in adverse birth outcomes between infants born to Black and White mothers within the same ICE quintile. For both outcomes, while the White–Black difference in these outcomes was consistently narrowest in the highest ICE quintile, even in these counties the difference was very large in magnitude. For example, the White–Black difference in preterm birth was −3.5 percentage points in 2018, with the rate for infants born to Black mothers 55% higher than that for infants born to White mothers. Thus, for both measures of infant health, infants born to Black mothers consistently experienced worse outcomes than those born to White mothers, regardless of the relative advantage of their community, as measured by ICE.

We build on this finding in Table 1 and assess the extent to which our proxy measures for structural racism explain the disparity in birth outcomes between infants born to White and Black mothers. The first column of Table 1 presents the total unadjusted difference in outcomes between infants born to White and Black mothers. Model 1 presents the difference in outcomes between infants born to Black and White mothers after accounting for ICE quintiles. Model 2 adds indicators of NDI quintiles. Model 3 adds state of residence indicators. Finally, model 4 adds individual-level maternal characteristics.

**Table 1.** Investigating potential mediating factors of the White–Black difference in birth outcomes, 2007–2018.

	Unadjusted model	Model 1	Model 2	Model 3	Model 4
Preterm birth	0.046^a^ (0.001)	0.042^a^ (0.001)	0.042^a^ (0.001)	0.042^a^ (0.001)	0.021^a^ (0.001)
Low birth weight	0.061^a^ (0.001)	0.058^a^ (0.001)	0.057^a^ (0.001)	0.058^a^ (0.001)	0.036^a^ (0.002)
ICE quintile	N	Y	Y	Y	Y
NDI quintile	N	N	Y	Y	Y
State of residence indicators	N	N	N	Y	Y
Maternal characteristics	N	N	N	N	Y

Abbreviations: ICE, Index of Concentration at the Extremes; N, no; NDI, Neighborhood Deprivation Index; Y, yes.

Source: 2007–2018 Vital Statistics Natality Files. ICE quintiles were constructed from 2006–2010 American Community Survey files, designating advantaged populations as non-Hispanic White households with incomes in the top income quintile and disadvantaged populations as non-Hispanic Black households with incomes in the bottom income quintile. NDI quintiles for 2006–2010 collected from Buller^33^ (see https://cran.r-project.org/web/packages/ndi/vignettes/vignette.html). Preterm birth was defined as births with obstetric estimates of gestational length at or earlier than 37 weeks. Low birth weight was designated based on birth weight <2500 g. All regressions include indicators for birth year. Maternal characteristics include mother's age, indicators for parity (first birth vs second or later birth), education (high school degree or less education, some college, Bachelor’s or greater education, and education status missing indicators), and marital status (married, not married, and marital status missing indicators). Standard errors are clustered at the county level and are reported in parenthesis.

^a^Indicates that the estimated White–Black difference in preterm birth or low birth weight is statistically significant at the *P* < .05 significance level.

Infants born to White mothers were 4.6 percentage points less likely to be born preterm and 6.1 percentage points less likely to be low birth weight than infants born to Black mothers. Including indicators for ICE quintiles (model 1) barely moved these estimates for either outcome, indicating that structural racism, as proxied for by ICE, does not meaningfully explain differences in birth outcomes between infants born to Black and White mothers. Further controlling for neighborhood deprivation measured at the county level (model 2) continued to have very little effect on the White–Black difference. Adding state of residence (model 3) also had almost no explanatory power for the total unadjusted White–Black difference for both outcomes.

Including maternal-level factors (model 4) had the most meaningful effect—slightly narrowing the White–Black disparity in preterm birth and low birth weight. The difference in preterm birth fell by 2.1 percentage points and the difference in low birth weight decreased by 2.2 percentage points, suggesting that these individual-level factors contribute to the overall White–Black disparity. While these are large relative decreases in each of the differences, the magnitude of the adjusted disparities in birth outcomes remains substantial (2.1 and 3.6 percentage points higher likelihood of preterm birth and low birth weight among infants born to Black mothers). Our analysis in Table 1 indicates that none of the measures we explored—ICE, NDI, state of residence, maternal age at birth, parity, education, and marital status—readily explain a substantial amount of the White–Black disparity in birth outcomes between infants born to White and Black mothers.

Table 2 presents the results of our decomposition analysis. The first and second columns report rates of each outcome among infants of White and Black mothers, respectively, and the third column reports the total White–Black difference. Decomposition results are presented in the last 3 columns of the table.

**Table 2.** Decomposition of 2018 White–Black differences in birth outcomes.

	Rate among White infants	Rate among Black infants	White-Black difference	Portion of White–Black difference attributed to differences in observed factors	Portion of White-Black difference attributable to differences in the effects of observed factors
Preterm birth	7.1%	11.7%	−4.6 pp	−2.5 pp^a^	−2.1 pp^a^
Low birth weight	5.1%	11.2%	−6.1 pp	−2.5 pp^a^	−3.6 pp^a^

Abbreviations: ICE, Index of Concentration at the Extremes; NDI, Neighborhood Deprivation Index; pp, percentage point.

Source: 2007–2018 Vital Statistics Natality Files. ICE quintiles were constructed from 2006–2010 American Community Survey files, designating advantaged populations as non-Hispanic White households with incomes in the top income quintile and disadvantaged populations as non-Hispanic Black households with incomes in the bottom income quintile. NDI quintiles for 2006–2010 collected from Buller^33^ (see https://cran.r-project.org/web/packages/ndi/vignettes/vignette.html). Preterm birth was defined as births with obstetric estimates of gestational length at or earlier than 37 weeks. Low birth weight was designated based on birth weight <2500 g. Predicted rates derived from regressions restricting to infants born to White mothers for each outcome on ICE quintile indicators, NDI quintile indicators, indicators for birth year, indicators for state of residence, and the following individual characteristics: mother's age, indicators for parity (first birth vs second or later birth), education (high school degree or less education, some college, Bachelor’s or greater education, and education status missing indicators), and marital status (married, not married, and marital status missing indicators).

^a^Indicates that shares of the overall differences attributable to observed factors, returns, or interactions are statistically significant at the *P* < .05 level.

The overall White–Black difference in rates of preterm birth is estimated at −4.6 percentage points (Table 2, column 3). Of this difference, −2.5 percentage points (or ∼54%) is attributable to differences between groups in ICE measures, NDI measures, state of residence, maternal age, birth parity, maternal education, and maternal marital status. Approximately −2.1 percentage points of the overall difference (or ∼46%) is attributable to differences in the effects of these factors experienced by births to White mothers relative to Black mothers.

The overall White–Black difference in low birth weight was −6.1 percentage points. Approximately 41% of this difference (−2.5 percentage points) is attributable to White–Black differences in ICE, NDI, geographic, and maternal characteristics. A larger share (−3.6 percentage points or 59%) is attributable to differences in the effects of these factors.

Altogether, the analysis in Table 2 indicates that the share of the overall White–Black difference in preterm birth attributable to differences in the effects of observable factors is, at a minimum, as relevant as the difference in the observed factors between themselves.

## Discussion

Our paper documents persistent disparities in birth outcomes between infants born to Black and White mothers between 2007 and 2018, from right after the Great Recession to just before the coronavirus pandemic. Consistent with several single-state studies, we found that infants born to Black mothers residing in areas with the highest levels of structural racism, as measured by the level of racialized disadvantage, are more likely to have infants born preterm and with low birth weights than those infants born to mothers residing in areas with the lowest levels of structural racism.^16,17,38^ The same is true for infants born to White mothers; however, infants born to Black mothers residing in areas with the lowest levels of racialized economic disadvantage are still more likely to be born preterm and with low birth weight than infants born to White mothers residing in areas with the highest levels of racialized economic disadvantage. Our findings are consistent with work that documents similar patterns by mothers' family income.^4^ These White–Black disparities in birth outcomes exist within every level of ICE, with the largest differences generally being in disadvantaged counties and the smallest in advantaged counties.

Numerous scholars have argued that structural racism is a fundamental cause of health disparities.^14,20,39,40^ Assessing the impact of structural racism on health inequities is challenging because of its pervasive role in US society.^40^ As conceptualized by Jones^13^ in 2000, 3 distinct levels of racism cause racial differences in health outcomes: institutionalized racism (such as the structures and policies that disadvantage people of color and advantage White people across factors associated with health), personally mediated racism (such as health care provider bias), and internalized racism (such as negative self-perception).

This paper has focused on the structural aspects of racism as measured by county-level ICE and NDI. Measurement of structural racism itself is challenging and measures can vary from area-level measures of both racial, economic, and residential advantage (including ICE, dissimilarity indices, and multiple proportions) to surveys of individuals' experience of discrimination or microaggression, to historical and contemporary policies.^35,40-42^ In multivariate models, area-level measures of structural racism such as ICE capture the association between economic and racial advantage and health, but also other aspects of structural racism correlated with these measures.^35^ In fact, when we include county-level measures of neighborhood deprivation, we see consistent levels of unexplained disparities in our outcomes as when they are not included. It is only when we include individual characteristics of the mothers (which are themselves influenced by structural racism) that the differences in outcomes for infants born to Black and White mothers are diminished.

White and Black mothers live in areas with different levels of structural racism and advantages and have different levels of education and rates of marriage, as well as different distributions of age and parity. Each of these factors is strongly associated with birth outcomes. Through our decomposition analysis, we see that differences in our observed factors of ICE, NDI, state of residence, and mother's characteristics account for just over half of the share of the differences in preterm birth and about 40% of the share of the differences in rates of low birth weight. A similar share of the White–Black difference in preterm birth and a greater estimated share of the White–Black difference in low-birth-weight infants are attributable to differences in the effects of these factors for infants of Black mothers relative to infants of White mothers. That is, when considering the same county ICE and NDI levels, state of residence, and maternal characteristics, we found that residing in a neighborhood with less disadvantage or less deprivation, or being born to a mother with greater educational attainment, is more likely to result in non-adverse birth outcomes for White mothers and their infants than for Black mothers and their infants. These findings are consistent with the work of Assari^43^ and others across a variety of health outcomes.

The fact that factors such as residing in a more advantaged neighborhood confer a lower benefit to Black mothers and infants than to White mothers and infants has implications for what policy solutions are required to achieve health equity. Health inequities are produced by structural racism’s multisystem impacts and solutions cannot be left only to the health care system. Our findings indicate that providing Black people with the observable advantages of White people is not enough to achieve equity in birth outcomes. Thus, transfer policies that are designed to shift resources to disadvantaged communities (potentially to match the resource levels of White people) are likely to narrow White–Black disparities in infant outcomes but are insufficient in eliminating these disparities altogether. Instead, policies need to target multiple systems and be tailored to the specific needs of Black people and other people of color. Policies expanding housing assistance in amenity-rich neighborhoods, for example, may have limited impact on White–Black infant health disparities; understanding why Black infant outcomes likely improve because of such policies but White–Black disparities do not narrow remains an important area for future research.

The health care system is not, however, without a broad and immediate role. Despite gains in coverage following the ACA, Black women of reproductive age are more likely than their White peers to be uninsured and 40% live in the 10 states that have not expanded Medicaid under the ACA, which contributes to this inequity. This limits their access to health care prior to becoming pregnant.^44^ Among those who are insured, nonelderly Black adults are more likely than their White peers to be covered by Medicaid and are, therefore, disproportionately disadvantaged by state policies that pay lower rates of reimbursement under the Medicaid program than other payers.^45,46^ At the community level, Black people are more likely to reside in primary care shortage areas, limiting their access to quality health care.^29^ Interpersonally, the health care system has a long history of discrimination that remains today. Increasing the number of providers of color and providing Black clinicians with enhanced support would offer more culturally appropriate care and enhance communication between patients and providers.^47^ Finally, as the system on the front lines of observing and treating disparities in health outcomes, the health care sector plays a central role in galvanizing multisystem support to address health inequities.^48^

## Supplementary Material

qxae092_Supplementary_Data
