# Supplementary material for: How structural racism, neighborhood deprivation, and maternal characteristics contribute to inequities in birth outcomes
Source: Health Aff Sch. 2024 Jul 23;2(8):qxae092. doi: 10.1093/haschl/qxae092 (PMC11296672; doi:10.1093/haschl/qxae092)
Supplement: qxae092_Supplementary_Data [file qxae092_supplementary_data.zip › supplementary-materials-Revision.docx]

**Supplementary Figure Legends**

**Appendix Figure 1. Distribution of ICE and NDI county-level estimates**

Source: ICE quintiles constructed from 2006-2010 American Community Survey files, designating advantaged populations as non-Hispanic white households with incomes in the top income quintile and disadvantaged populations as non-Hispanic Black households with incomes in the bottom income quintile. NDI quintiles collected from Buller (2023), see: https://cran.r-project.org/web/packages/ndi/vignettes/vignette.html.

**Appendix Figure 2. 2007-2018 Share of Births Born Preterm or with Low Birthweight among Infants Born to Black and White Mothers.**

Source: 2007-2018 Vital Statistics Natality Files.

Notes: Preterm birth defined as births with obstetric estimates of gestational length at or earlier than 37 weeks. Low birthweight designated based on birth weight <2,500 grams.

| **Appendix Figure 1. Distribution of ICE and NDI county-level estimates** | |
| --- | --- |
| **ICE** | **NDI** |
| 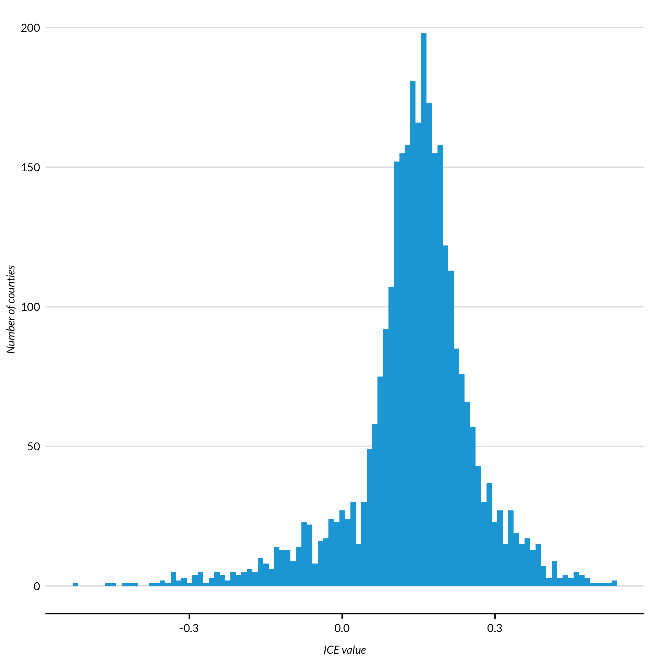 | 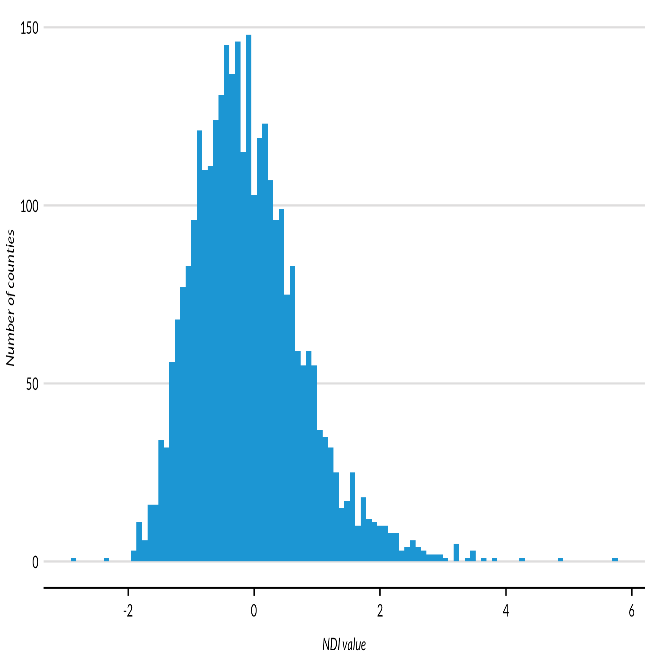 |
| Source: ICE quintiles constructed from 2006-2010 American Community Survey files, designating advantaged populations as non-Hispanic white households with incomes in the top income quintile and disadvantaged populations as non-Hispanic Black households with incomes in the bottom income quintile. NDI quintiles collected from Buller (2023), see: https://cran.r-project.org/web/packages/ndi/vignettes/vignette.html. | |

| **Appendix Figure 2. 2007-2018 Share of Births Born Preterm or with Low Birthweight among Infants Born to Black and White Mothers.** | |
| --- | --- |
| 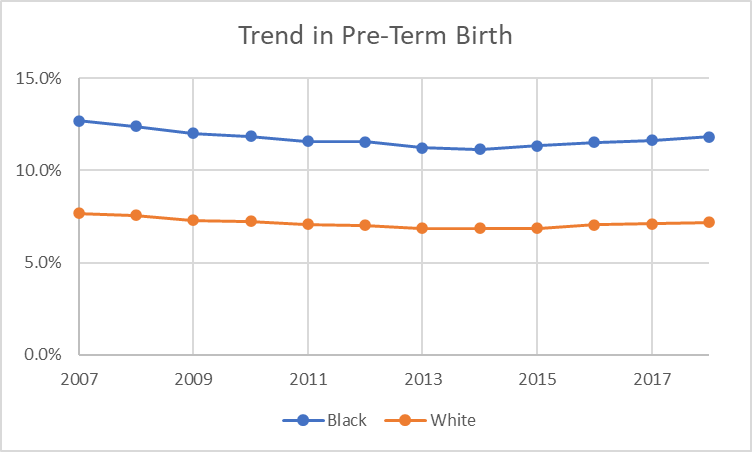 | 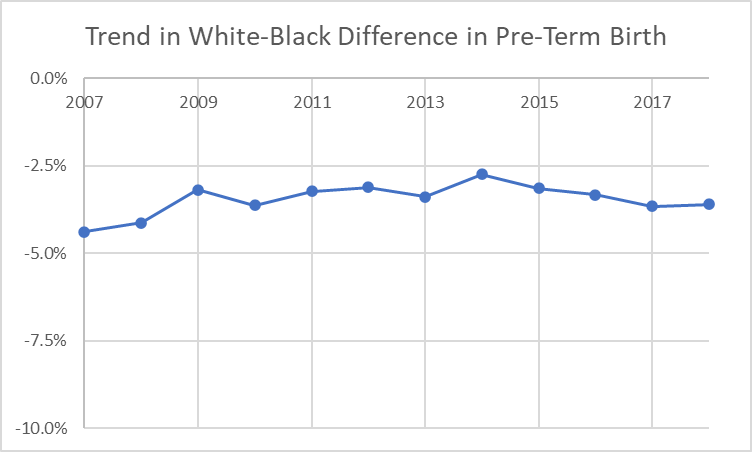 |
| 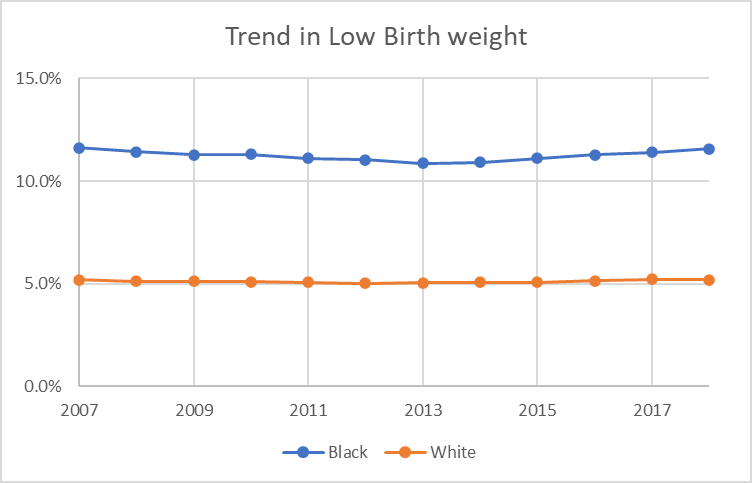 | 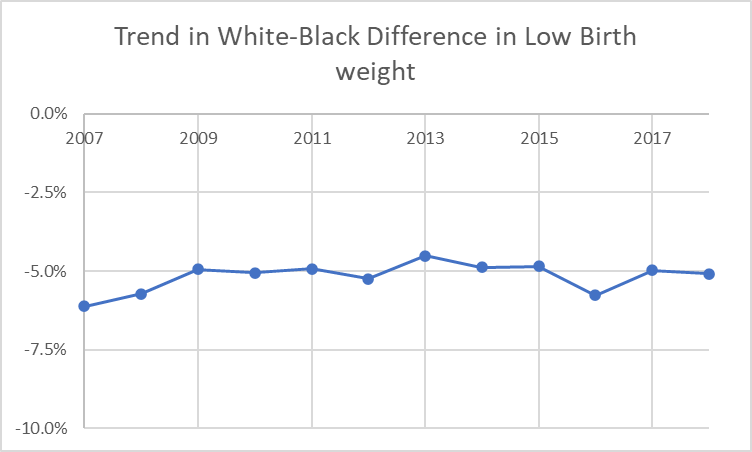 |
|  |  |
| **Source:** 2007-2018 Vital Statistics Natality Files.  **Notes:** Preterm birth defined as births with obstetric estimates of gestational length at or earlier than 37 weeks. Low birthweight designated based on birth weight <2,500 grams. | |
